# The long‐term outcome and changes in tricuspid regurgitation pressure gradient in dogs diagnosed with pulmonary hypertension and *Angiostrongylus vasorum* infestation

**DOI:** 10.1111/jsap.13893

**Published:** 2025-06-27

**Authors:** R. Turner, D. Connolly, D. Brodbelt, S. Cortellini

**Affiliations:** ^1^ Department of Clinical Science and Services The Royal Veterinary College, University of London Hatfield UK; ^2^ Department of Pathobiology and Population Sciences Royal Veterinary College Hatfield UK

## Abstract

**Objectives:**

Angiostrongylus vasorum (AV) is a metastrongylid parasite that has been associated with pulmonary hypertension (PH) in dogs. The objectives of the study were to describe the clinical presentation of dogs with AV and PH, document changes in tricuspid regurgitation maximum pressure gradient (TR Max PG) in subsequent months and years, record the survival to discharge and report the long‐term survival of these dogs and factors associated with mortality.

**Materials and Methods:**

Data from client‐owned dogs presenting to a teaching hospital between January 2007 and October 2023 with AV and PH were reviewed retrospectively. Signalment, presenting signs and echocardiographic reports were collected, and their survival to discharge noted. Date of death and loss of follow‐up were recorded. Univariable analysis was used to assess the association of different factors on long‐term survival.

**Results:**

Twenty‐eight cases were identified with concurrent PH and AV, commonly presented in respiratory distress. Tricuspid regurgitation, as measured by TR Max PG on echocardiography, resolved in 9 of 28 (32.1%) cases. Survival to discharge was favourable at 92.9% (26/28). The median duration of follow‐up was 196 days. Survival time was documented, with 6 of 11 (54.5%) known dogs still alive at 2 years post discharge. Treatment with sildenafil (Viagra; Pfizer) was associated with longer survival time and increased age was associated with a shorter survival time. The presence of right‐sided congestive heart failure was not associated with a shorter survival time.

**Clinical Significance:**

Dogs with AV infestation and PH can live for prolonged periods (>2 years).

## INTRODUCTION

*Angiostrongylus vasorum* (AV) is a metastrongylid parasite that can cause a range of clinical signs in the domestic dog. The parasite has an indirect lifecycle with canids as definitive hosts and molluscs as intermediate hosts (Bolt et al., 1994; Koch & Willesen, 2009; Morgan et al., 2010). *Angiostrongylus vasorum* infestation often presents in dogs as either cardiorespiratory signs, haemorrhagic diathesis or neurological signs. Clinical signs range from mild, such as lethargy, anorexia, vomiting or diarrhoea, to more severe signs of coughing, tachypnoea and dyspnoea in cases with cardiopulmonary involvement (Chapman et al., 2004; Colella et al., 2016). Reported mortality rates range from 2% to 13% (Helm & Morgan, 2017; Morgan et al., 2005). A recent study suggests that those with clinical bleeding diathesis are associated with poorer long‐term survival rates (Thomsen et al., 2024). Pulmonary hypertension (PH) can develop and has been reported in up to 14.5% of cases of AV and dogs with both AV and PH have a poorer survival time than those with AV without PH (Borgeat et al., 2015). *Angiostrongylus vasorum* infestation can lead to severe pulmonary parenchymal changes such as thrombosis, haemorrhage, inflammation, periarteritis and coalescing granulomata. These changes can lead to pulmonary hypertension (Glaus, 2013; Matos et al., 2012; Reinero et al., 2020).

Pulmonary hypertension is defined as increased pulmonary pressure within the pulmonary vasculature (Nicolle et al., 2006). Pulmonary hypertension arising from parasitic disease is one of the six categories of PH: (1) pulmonary arterial hypertension, (2) left heart disease, (3) respiratory disease and hypoxia, (4) pulmonary emboli, pulmonary thrombi, pulmonary thromboemboli, (5) parasitic disease and (6) disorders that are multifactorial or with unclear mechanisms (Glaus, 2013; Reinero et al., 2020). Changes associated with PH can be identified in three main sites: the ventricles, the pulmonary artery and the right atrium/ caudal vena cava. Ventricular changes include flattening of the interventricular septum, underfilling or decreased size of the left ventricle, right ventricular hypertrophy and right ventricular systolic function. The pulmonary artery (PA) changes include PA enlargement, reduced distensibility index, reduction in right ventricular outflow Doppler acceleration time or acceleration time to ejection ratio or presence of systolic notching of the Doppler RV outflow profile. Right atrium and right caudal vena cava enlargement may also be present. Clinical guidelines for diagnosis and management of PH (Reinero et al., 2020) have suggested classification of the probability of PH as low, intermediate or high using the peak tricuspid regurgitation velocity and the number anatomical sites affected as stratification criteria. Peak tricuspid regurgitation velocity (m/s) can be measured via Doppler echocardiography and the pressure gradient is calculated using the simplified Bernoulli equation to obtain the tricuspid regurgitation maximum pressure gradient (TR Max PG), expressed as Pressure gradient (mmHg) = 4 × *V* ^2^ where *V* represents the tricuspid regurgitation velocity (m/s).

There are limited data to guide practitioners on the management of PH resulting from AV, as the clinical responses to pharmacological treatment appear to be variable (Borgeat et al., 2015). One study suggested that chronic infestation of AV may be important for the development of PH as acutely infested dogs did not develop PH (Kranjc et al., 2010). Several case reports have documented resolution of PH post treatment of both AV and PH (Estèves et al., 2004; Nicolle et al., 2006). In contrast, a recent case report describes a patient with AV and irreversible pulmonary hypertension. The patient presented with abdominal distension and ascites. After initial treatment with oral furosemide and oral milbemycin oxime with praziquantel, the patient was referred and diagnosed with AV and PH. After further treatment, to which they responded, the ascites recurred. This patient had ruptured chordae tendineae of the tricuspid valve, leading to volume and pressure overload (Szatmári, 2020). Median survival times have not been described, and no studies have reported the overall progression or resolution of PH for naturally infested dogs in a larger cohort of patients.

The aim of this study was to evaluate the survival after diagnosis of AV and PH. The objectives of the study were to (1) describe the clinical presentation of dogs with AV and PH, (2) document changes in TR Max PG by Doppler echocardiographic measurement in subsequent months and years, (3) record the survival to discharge for these dogs and finally (4) report the long‐term survival of these patients and factors associated with mortality.

## MATERIALS AND METHODS

**FIG 1:**
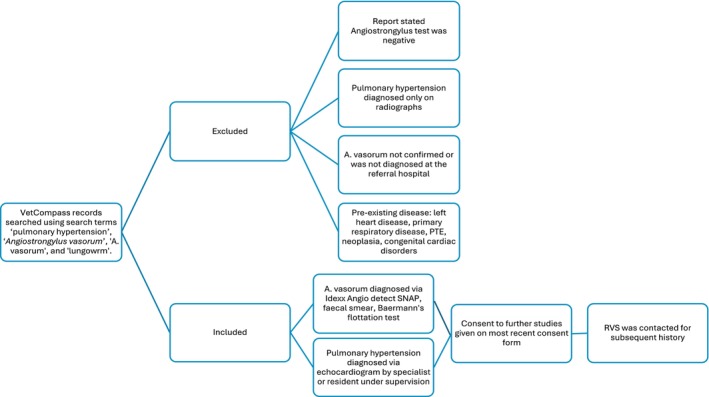
Diagram depicting inclusion and exclusion criteria for those that presented at the Royal Veterinary College with Angiostrongylus vasorum infestation and pulmonary hypertension.

This was a retrospective cohort study with client‐owned dogs naturally infested with AV and followed to determine survival after diagnosis of PH. Ethical approval from the Social Sciences Research Ethical Review Board at the Royal Veterinary College (SSRERB URN SR2023) was obtained. An institutional data silo (VetCompass database) was used to search for all dogs diagnosed with AV and PH from January 2007 to October 2023 using the key words ‘Angiostrongylus’, ‘*A. vasorum*’, ‘lungworm’ and ‘pulmonary hypertension’ at a teaching hospital, the Queen Mother Hospital for Animals, Royal Veterinary College. Data were initially extracted on 27 July 2022, and additional cases were searched for on 17 November 2023. The cases retrieved were imported into a Microsoft excel spreadsheet which included a unique patient identification number, sex, neuter status, breed and age at presentation. These cases were manually reviewed by a single operator (RT) by using the identification number in the hospital’s clinical record system to determine eligibility (Fig 1).

*Angiostrongylus vasorum* was diagnosed by an Angio detect™ antigen test, a positive faecal smear and/or a positive Baermann’s test performed at the institution. Pulmonary hypertension was diagnosed if an echocardiography by a cardiology specialist or specialist in training under supervision recorded a TR Max PG of greater than 46 mmHg, which gives intermediate (if no additional supporting anatomical sites abnormalities) or high (if one or more anatomical sites abnormalities present) probability of pulmonary hypertension (Reinero et al., 2020). If tricuspid regurgitation was not present on colour Doppler assessment, then a value of zero for TR Max PG was assigned for statistical analysis. Cases were included if both these criteria were met and for these cases, referring veterinarians were contacted if the client had opted into further studies on their most recent consent form.

Cases were excluded if PH was diagnosed by radiographic imaging only, if the diagnosis of AV was not performed at the referral hospital or was reported as negative. Full echocardiograms were reviewed to exclude alternative causes for increased right ventricular pressure (e.g. pulmonic stenosis) or other causes for PH, including left‐sided heart disease which may result in post‐capillary PH. Clinical records, including further diagnostic imaging, were reviewed to exclude other pre‐existing primary respiratory disease, pulmonary thromboembolic disease associated with other pro‐coagulant conditions, intrathoracic neoplasia or congenital cardiac disorders which might contribute to PH (Kellihan & Stepien, 2010).

Data extracted included breed, sex, neuter status, age and clinical signs on presentation, treatment received at time of diagnosis, TR Max PG on the first visit and subsequent visits to the institution, duration of hospital visit at first diagnosis, survival to discharge and time of loss of follow‐up.

It was recorded if coughing was a presenting complaint. Data retrieved from clinical record at presentation included heart rate, rectal temperature, respiratory rate, whether increased respiratory effort was perceived by the attending clinician and Doppler systolic blood pressure. Presence of neurological deficits were also recorded if a neurological exam was performed and described as abnormal. Presence of right sided congestive heart failure (rCHF) was also recorded if diagnosed by a cardiologist based on clinical signs such as hepatic venous congestion and ascites and echocardiographic evidence of PH (Schober et al., 2010). Patients were classified as “coagulopathic” if clinical signs consistent with bleeding diathesis were present and thromboelastograph (TEG) (TEG 5000, Haemonetics Corp.) and/or Viscoelastic coagulation monitoring (VCM®) and/or prothrombin (PT) and activated partial thromboplastin time (aPTT) (Coag DX analyser, IDEXX) tests had been performed.

Treatment was recorded for each case including if they received imidacloprid and moxidectin (Advocate; Boehringer Ingelheim) and a course of fenbendazole (Panacur; Intervet). The dose and duration of the fenbendazole course were recorded. It was also recorded if they had been started on sildenafil (Viagra; Pzifer), and the dose and duration of this.

The echocardiography reports were manually reviewed. The trend of TR Max PG (mmHg) changes was plotted over time for each patient where at least two echocardiographic examinations were performed. The time (in days) between each echocardiographic examination was recorded. If a repeat test for AV was performed at either the referring veterinary surgery or at the authors’ institution, then this was recorded to assess the resolution of AV.

Descriptive statistics were performed in R (version 4.4.0, 2024‐04‐24) using RStudio (version 2024.04.0 + 735) (R Core Team, 2024). Each numerical data set was tested for normality using a Shapiro–Wilks test. Parametric groups were described using the mean and standard deviation (SD), whereas non‐parametric data sets were described using the median and interquartile range (IQR).

If dogs survived to discharge, the duration of their initial hospital visit was recorded. It was recorded if they died naturally or were euthanased during hospitalisation. The date of discharge was noted, and the clinical records were reviewed to identify the last known update on the dogs’ status. The time to death, last record or loss of follow‐up was recorded. For those dogs whose owners consented to participation in further studies, the referring veterinary practices were contacted to obtain further clinical history. If dogs had died, their time to death was recorded, and the cause of death was recorded if available. Survival time was defined as the time from discharge to the date of death.

A Kaplan–Meier curve was used to represent survival time. A log‐rank test was used to assess the association of several factors individually on survival time (Dohoo et al., 2010). These included age (two binary variables: over the median age and less than the median age), sex, neuter status, presence of coughing, if the patient was in rCHF, if treated with sildenafil or not and the TR Max PG (at both the first and second echo). Tricuspid regurgitation maximum pressure gradients were categorised into those below the median TR Max PG on presentation and those equal to or greater than the median TR Max PG on presentation. Statistical significance was set at the 5% level.

## RESULTS

### Signalment

In total, the medical records from 414 cases were manually reviewed and assessed for eligibility. Twenty‐eight cases were identified with concurrent AV infestation and evidence of increased TR Max PG (over 46 mmHg). Causes for exclusion were negative AV test, AV test not performed at the referral hospital, no echocardiography performed, left‐sided heart disease, pulmonary thromboembolic disease, intrathoracic neoplasia and congenital cardiac disorders.

Table 1 summarises the percentage of cases according to sex, neuter status and breed. The youngest patient was 5 months old; the oldest was 14 years and 11 months. The median age on presentation was 3 years and 7 months (IQR: 11 months to 8 years and 7 months).

**Table 1:** Summary of sex, neuter status, and breeds in cases of AV and PH

Signalment	Number of cases and percentage of total cases (total = 28)
Sex and neuter status	
Female entire	2 (7.1%)
Female neutered	5 (17.8%)
Male entire	15 (53.6%)
Male neutered	6 (21.4%)
Breed	
Yorkshire terrier	3 (10.7%)
Cavalier King Charles Spaniel	3 (10.7%)
Jack Russell Terrier	3 (10.7%)
Staffordshire Bull Terrier	2 (7.1%)
Miniature dachshund	2 (7.1%)
Pug	2 (7.1%)
Border Collie	1 (3.6%)
Bulldog	1 (3.6%)
Chihuahua	1 (3.6%)
Cockapoo	1 (3.6%)
Cocker spaniel	1 (3.6%)
Dachshund	1 (3.6%)
Golden retriever	1 (3.6%)
Labrador	1 (3.6%)
Mixed breed	1 (3.6%)
Toy poodle	1 (3.6%)
Welsh Terrier	1 (3.6%)
West Highland White Terrier	1 (3.6%)
Yorkshire terrier cross	1 (3.6%)

### Clinical presentation

Table 2 shows the triad of clinical signs that AV patients presented with and the proportion of cases within these categories. Coughing was recorded if it was part of the presenting complaint. The dog who had neurological signs also presented with signs of respiratory distress. This dog had a right‐sided head turn, non‐ambulatory tetraparesis and falling to the right. There were reduced postural reactions on the left pelvic limb and decreased menace in the right eye, with a neurolocalisation to the right forebrain. Three dogs were coagulopathic; these dogs all had increased respiratory rate, and two had increased effort. One dog presented with a recent history of syncope but had no increased respiratory rate or effort, coagulopathy or neurological deficits. Table 3 shows the initial vital signs on presentation. Eleven (39.3%) dogs presented in rCHF.

**Table 2:** Triad of clinical signs in dogs and their overall representation

Clinical signs	Number of cases and percentage of total cases (total = 28)^†^
Cardiorespiratory signs	
Increased respiratory effort	19 (67.8%)
Coughing	10 (35.7%)
Increased respiratory rate	24 (85.7%)
Neurological signs	1 (3.6%)
Coagulopathy	3 (10.7%)

^†^ Dogs can present with more than one of these signs.

**Table 3:** Physical examination findings with mean and standard deviation

Physical examination findings	Mean (SD)^†^
Heart rate	142 beats per minute (35)
Temperature	38.2°C (0.6)
Respiratory rate	60 breaths per minute (22)
Systolic non‐invasive Doppler blood pressure	133 mmHg (29)

^†^ All physical examination findings were normally distributed and thus the mean and standard deviation are represented here.

### Treatment

All 28 dogs were initially treated for AV with imidacloprid and moxidectin, followed by a course of fenbendazole. The duration of fenbendazole treatment varied among individuals (Table 4). The median dose of fenbendazole was 50 mg/kg (IQR: 49.8 to 51.4) over a median length of 10 days (IQR: 7 to 10). One case lacked confirmation of the fenbendazole dose, and in five cases, the duration of the course could not be obtained from the clinical records.

**Table 4:** Description of initial sildenafil dose, dosing regimen, fenbendazole dose and course length at initial treatment

Total daily sildenafil dose (mg/kg/24 hr)	Sildenafil timing^†^	Fenbendazole dose (mg/kg)	Fenbendazole course length (days)
–	–	50	7
8	BID	50	10
7.5	TID	–	–
1.34	BID	47	10
–	–	52	10
2.8	BID	50	7
–	–	50	10
3.4	BID	55	7
1.2	BID	48	–
1.9	SID	49.6	–
1.08	BID	54	
1.34	BID	27	–
5.6	BID	50	10
4.4	TID	51.7	7
3.72	TID	42.9	7
6	TID	79	7
8.61	TID	48	7
3	TID	50	10
7.8	TID	50	10
2.25	TID	50	5
3.9	TID	53.5	14
5.1	TID	54	10
8.7	TID	50	10
3.52	BID	46.9	14
6	TID	50	20

^†^ SID, BID and TID refer to the frequency of dosing of either once a day, twice a day or three times a day, respectively.

Twenty two of the 28 dogs were treated with sildenafil to manage PH. The dose, interval and course duration were variable (Table 4). The median total daily dose of sildenafil was 3.72 mg/kg (IQR: 2.1 to 6.3). The ongoing treatment regimen of sildenafil was not clear from the retrospective data, however, there were eight dogs where treatment was advised to be discontinued due to resolution of PH. Of those who survived to discharge, only 4 were not treated with sildenafil. It is not clear from clinical notes why dogs were not prescribed sildenafil; in two dogs there were no comments, in one patient they were initially treated with fenbendazole alone and 2 months later with no improvement of PH, sildenafil was then commenced. In the final dog, clinical records suggested that it was opted not to treat with sildenafil as severity of PH was low, however, “sildenafil could have been considered in the future”; there are no further communications regarding this dog. These dogs were treated early in the data collection period, with the most recent discharge occurring in 2017.

### Echocardiographic assessment

On presentation, the median TR Max PG was 88.9 mmHg (IQR: 75.0 to 97.8) in all dogs presenting with PH and AV. Of the dogs that survived to discharge (*n* = 26), 25 dogs had at least two echocardiograms recorded at the hospital. In total, 79 discharge reports were reviewed along with associated echocardiographic reports if available. For those that survived to discharge, there was documented evidence of a negative AV test at subsequent visits in 18 of 26 cases (69.2%).

**FIG 2:**
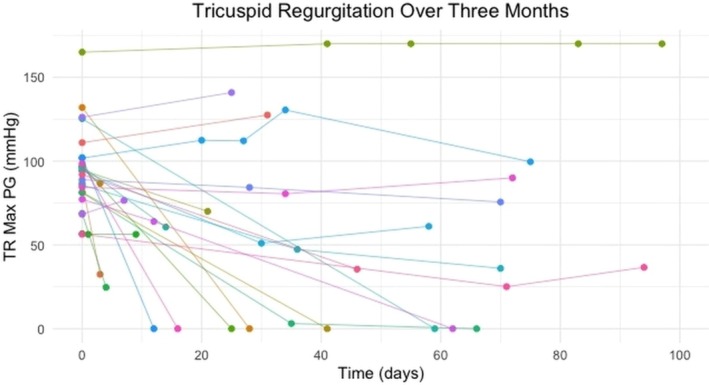
A representation of the TR Max PG for each dog in a 3‐month period. Each line represents a different patient. Each dot represents the TR max PG recorded by echocardiography at the hospital.

There were six echocardiographic reports where TR Max PG (mmHg) was not available at follow‐up. The median time to TR Max being recorded as 0 (i.e. absence of visible tricuspid regurgitation on colour Doppler) was 41 days (IQR: 25 to 66) with the earliest being recorded at 12 days and the latest being recorded at 646 days. Fig 2 shows the changes in TR Max PG (mmHg) in the first 3‐month period post initial diagnosis.

**FIG 3:**
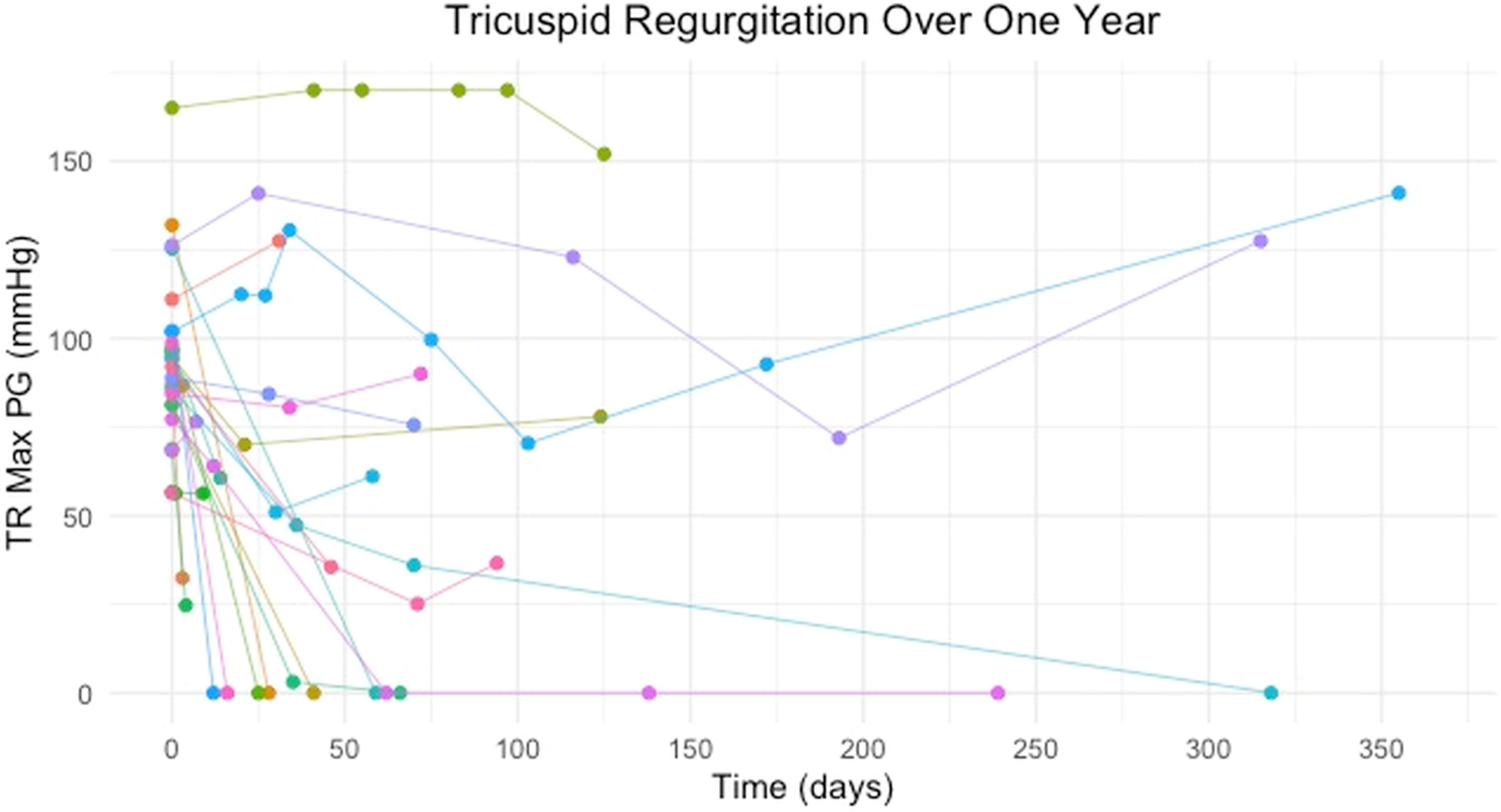
A representation of the TR Max PG for each patient in a 1‐year period. Each line represents a different dog. Each dot represents the TR max PG recorded by echocardiography at the hospital.

Each line in both Figs 2 and 3 shows whether a dog’s TR Max PG resolved, decreased, remained static or increased. Beyond 12 months, the patient status of increased, static, decreased and resolved remained the same apart from two dogs. One dog had resolution at 12 months, but there was mild evidence of tricuspid regurgitation at 512 days (TR Max PG = 28.7 mmHg; initially = 77.2 mmHg), so still below the threshold of PH in the absence of other anatomical signs. One dog who was static at 12 months had a decrease in TR Max PG after this time point.

Five other dogs had a reduction of TR Max PG to below 46 mmHg, but still measurable; 46 mmHg indicates a high probability of PH by Doppler echocardiography, even in the absence of any anatomical changes (Reinero et al., 2020). The TR Max PG of these dogs was as follows: 32.44 mmHg (initially: 86.95 mmHg), 24.69 mmHg (initially: 68.81 mmHg), 36 mmHg (initially: 85.75 mmHg), 35.52 mmHg (initially: 92 mmHg) and 36.6 mmHg (initially: 56.4 mmHg).

In summary, on follow‐up, no TR jet was seen on colour Doppler in 9 of 25 cases (35%), there was a decrease of the TR Max PG to below 46 mmHg in 10 of 25 (40%) dogs, 2 of 25 (8%) cases remained static and 4 of 25 (16%) had an increased TR Max PG.

### Hospitalisation and survival analysis

**FIG 4:**
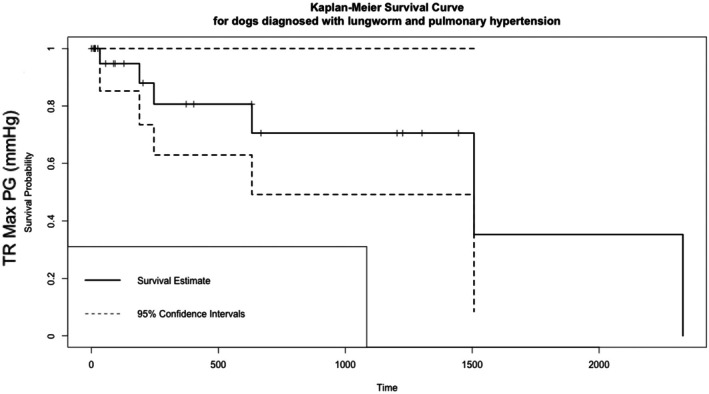
A Kaplan–Meier survival curve for dogs that were discharged. This was calculated from the discharge date to the date of death (*n* = 26). The solid line represents the estimated survival probability, with dashed lines indicating the 95% confidence intervals. Short, dashed marks on the horizontal segments represent censored observations.

The median duration of hospitalisation for those that survived to discharge was 3 days (IQR: 2 to 5), with a range of 1 to 14 days. Two dogs (2/28, 7.1%) did not survive to discharge from diagnosis. One had respiratory fatigue and was placed on mechanical ventilation but was also subsequently diagnosed with concurrent aspiration pneumonia with multidrug‐resistant bacterial infection and was not weaned off mechanical ventilation. The second dog suffered cardiopulmonary arrest while in the hospital, suspected due to underlying AV and the severity of PH; this dog was unable to be weaned off oxygen and the initial echocardiogram showed severe pulmonary hypertension and a TR Max PG of 119.77 mmHg. For those who survived, the median duration of follow‐up (time to death or to loss to follow‐up) was 196 days (IQR: 27 to 659) post discharge. Fig 4 outlines the long‐term survival of these dogs. Overall, of the 26 dogs that survived to discharge, the date of death was recorded for 6 dogs and the last known date they were alive was recorded for 20 dogs. Table 5 summarises the proportion of dogs that had died, been lost to follow‐up at 1 month, 6 months, 1 year and 2 years post discharge. There were insufficient deaths recorded to assess overall median survival time. The age of death was calculated for the 6 dogs where the date of death was known: 7 years and 6 months, 10 years, 5 years and 2 months, 14 years and 10 months, 11 years and 8 months, 11 years and 10 months.

**Table 5:** Survival to discharge at 1 month, 6 months, 1 year and 2 years, including status at follow‐up (alive, deceased or lost to follow‐up)

	1 month	6 months	1 year	2 years
Alive	19/19 (100%)	14/15 (93.3%)	11/14 (78.6%)	6/11 (54.5%)
Died	0/19 (0%)	1/15 (6.7%)	3/15 (20.0%)	5/11 (54.5%)
Lost to follow‐up	7/28 (25.0%)	11/28 (39.3%)	12/28 (42.9%)	15/28 (53.6%)

For the six dogs where a date of death was known, the cause of death was suggested to be related to AV and PH for two out of six dogs (33.3%) as stated in the clinical records. The cause was not known, but suspected to be related to PH and AV, in three dogs (50%) and one (16.7%) dog was euthanased at 2327 days due to “behavioural reasons” which may or may not have been related to ongoing AV infestation. Four of the six patients died naturally, and two were euthanased.

**FIG 5:**
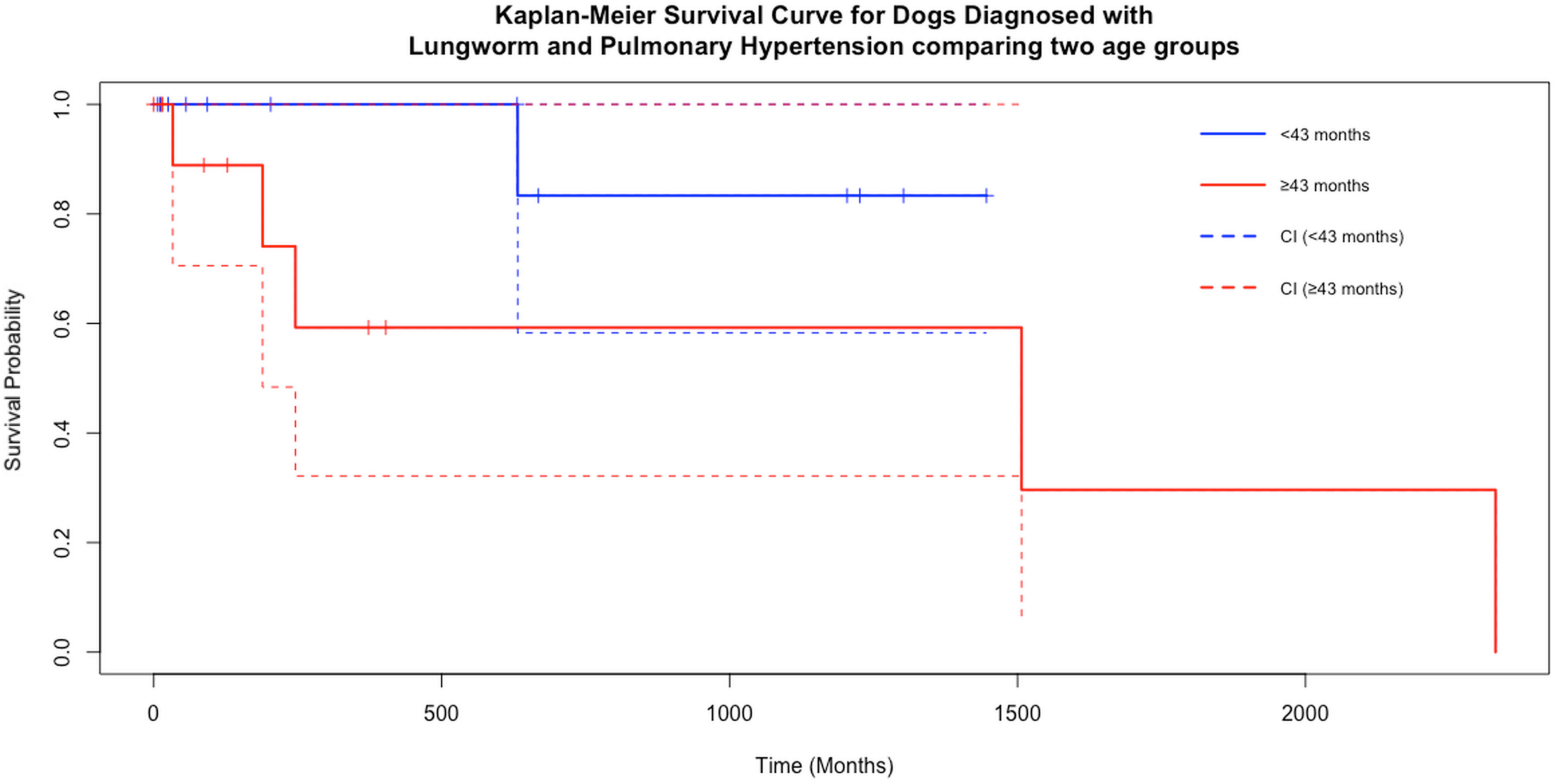
A Kaplan–Meier survival curve for dogs that were discharged, comparing the effect of being under the median age of 43 months *versus* equal to or over the median age. The solid lines represent the estimated survival probabilities, with dashed lines indicating the 95% confidence intervals. Short, dashed marks on the horizontal segments represent censored observations.

**FIG 6:**
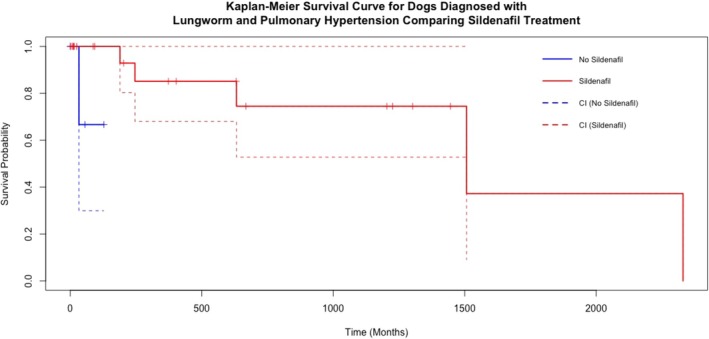
A Kaplan–Meier survival curve for dogs that were discharged, comparing the effect of being treated with Sildenafil or not. The solid lines represent the estimated survival probabilities, with dashed lines indicating the 95% confidence intervals. Short, dashed marks on the horizontal segments represent censored observations.

A log rank test was used to assess the association of several factors with those who survived to discharge. As shown in Table 6 and Fig 5, those that were over the median age were associated with poorer survival (P = 0.03). Being treated with sildenafil was associated with a longer survival time (P = 0.02), though the number not treated with sildenafil was small (*n* = 4) (Fig 6). No association with the dose of sildenafil was detected (P = 0.50).

**Table 6:** The association of different variables with survival

Category^†^	Log rank P value^‡^
Age	
<43 months old (*n* = 14)	0.03*
≥43 months old (*n* = 12)	
Sex (M = 20; F = 6)	0.09
Neuter status (Y = 10; N = 16)	0.50
Coughing on presentation (Y = 10; N = 16)	0.20
TR max PG at first echo	
<89 mmHg (*n* = 13)	0.50
≥89 mmHg (*n* = 13)	
TR max PG at second echo	
<89 mmHg (*n* = 19)	0.60
≥89 mmHg (*n* = 2)	
Presented in rCHF (Y = 11; N = 15)	0.90
Treated with sildenafil (Y = 22; N = 4)	0.02*
Sildenafil daily dose	
<3.72 mg/kg (*n* = 12)	0.50
≥3.72 mg/kg (*n* = 11)	

^†^ For numeric variables, these were categorised into binary variables: above the median or below the median of that variable.

^‡^ P value < 0.05.

* Shows statistically significant values.

There was no association between long‐term survival and sex, neuter status, presence of coughing, the severity of TR Max PG at both first and second echo and the presence of right‐sided CHF on presentation. Of the 11 dogs that presented in rCHF, 7 dogs had resolution of their rCHF (63.6%).

## DISCUSSION

This is the largest study to describe the long‐term outcome of dogs naturally infested with AV and diagnosed with PH. The main findings were that these dogs can live for prolonged periods (>2 years) and that survival was positively associated with sildenafil treatment. The presence of rCHF was not associated with a poorer outcome.

In the present study, male entire dogs represented most cases (15/28 (53.6%)). A previous study found male entire dogs to be overrepresented for contracting AV, but this was not a statistically significant risk factor for being clinically affected (Morgan et al., 2010), and this was looking at only AV infestation and not AV with PH. It may be that the behaviour of these dogs is more likely to make them at risk for contracting AV. Our finding that multiple breeds were affected supports previous literature suggesting that any breed can be affected by AV (Blehaut et al., 2014; Chapman et al., 2004; Elsheikha et al., 2014; Morgan et al., 2010).

The median age in this study at diagnosis was 48 months old (4 years old). Younger dogs have been seen to be at increased risk of infestation of AV alone in other study populations potentially as a result of lack of preventative medication or from being more playful in environments where the intermediate host is present (Chapman et al., 2004; Glaus, 2013). In contrast, older dogs may have a degree of acquired immunity or have different behaviours compared to younger dogs.

On presentation, the majority of the dogs were tachypnoeic with increased respiratory effort. This is the most common clinical sign for any patient that had a positive test result for AV (Glaus et al., 2010; Morgan et al., 2010). This observation suggests that PH should be screened for in all patients with cardiorespiratory signs with a positive AV test; although the cause of the tachypnoea and increased respiratory effort may be related to the parasitic burden itself and pulmonary parenchymal disease or PH.

Diagnosing pulmonary hypertension is complex, and a recent consensus statement has aimed to standardise the approaches for identifying and classifying PH (Reinero et al., 2020). Due to the absence of a specific diagnostic procedure that is both clinically feasible and capable of definitively confirming PH, a probabilistic approach has been recommended. Dogs with TR Max PG cut‐off of greater than 46 mmHg (TR velocity > 3.4 m/s) should be considered at intermediate or high probability of PH and likely to have the condition. In our study, we used the single Doppler echocardiographic parameter of TR Max PG to identify patients with AV‐associated PH and to monitor the progression of the disease. However, clinical findings, such as syncope, respiratory distress at rest, activity terminating in respiratory distress and right‐sided heart failure, are also strongly suggestive of PH (Reinero et al., 2020).

One of the biases inherent in retrospective studies is the lack of treatment standardisation, which may influence outcomes but cannot be evaluated for its impact on outcomes using this study methodology. The median fenbendazole dose was 50 mg/kg over 7 to 10 days, showing that there is some consistency among clinicians, in line with recommendations from a previous study (Elsheikha et al., 2014). However, there was less consistency with the dose and timing of sildenafil dose. One of the striking results was that patients treated with sildenafil had a significantly longer survival time than those untreated. However, among those who survived to discharge, only four did not receive sildenafil, resulting in a sample size too small for robust comparisons. The data collected in this study dates back to 2007 when the confidence in the use of sildenafil was lower due to concerns regarding its impact on systolic blood pressure. Over time, clinicians have become more comfortable in its use for PH. Indeed, since 2017, all the patients who survived to discharge were prescribed sildenafil.

Treatment with sildenafil has been shown to improve tricuspid regurgitation gradients and also be associated with the resolution of pulmonary alveolar infiltrates likely related to PH itself (Kellihan & Stepien, 2010).

Interestingly, recent PH management guidelines suggest that phosphodiesterase‐5 inhibitors (e.g. sildenafil) can be considered in the treatment of parasitic PH, although the current evidence available in AV‐associated PH does not support an association between sildenafil and survival (Borgeat et al., 2015; Reinero et al., 2020). The present study is the first to suggest an association, although there was not a significant dose‐related effect of sildenafil on survival. However, there was a wide total daily dose across the whole population, supporting the view that a prospective study would be warranted to assess the impact of phosphodiesterase‐5 inhibitors in the treatment of PH. A genetic polymorphism *(PDE5A:E90K)* in dogs, associated with lower cyclic guanosine monophosphate (cGMP) levels, has also been recently reported and is associated with decreased improvements in quality of life compared to wildtype dogs after sildenafil treatment; however, in this study, the PDE5A:E90K polymorphism was not assessed, as this is not routinely performed at the authors’ institution, nor is it widely available. Hence, the presence of the polymorphism could have influenced the response to treatment and outcome (Ueda et al., 2019). Matos et al. (2012) described recruitment of arteriovenous pulmonary shunts as detected using saline contrast echocardiography in four dogs who were experimentally infested with AV. Pulmonary arterial pressure (PAP) was measured invasively using right‐sided catheterisation. The two other dogs in the study did not recruit these shunts and developed higher PAP. These arteriovenous shunts could help decrease the risk of development of PH or reduce its severity (Matos et al., 2012). The current study would suggest that these patients did not develop these shunts, or they were poorly developed. However, our inclusion criteria were those that had PH and AV and did not include a population of dogs that had AV without PH. For further investigation into this hypothesis, CT angiography or saline contrast echocardiography could be performed in both populations for comparison.

Pulmonary hypertension is associated with pulmonary vascular constriction, hence vasodilatory agents (such as sildenafil) are effective. Chronic PH leads to a complex interaction of immune cells and vascular stromal cells (Tobal et al., 2021), resulting in muscularisation of vessel walls and endothelial cell proliferation, ultimately determining vascular remodelling. It is possible that the regulation of these remodelling mechanisms is related to the severity and reversibility of PH and could represent future therapeutic targets (Li et al., 2001; Sakao et al., 2010).

The degree of TR Max PG at either the first or second echo and presence of rCHF also had no statistical association with long‐term survival in this study. However, this is in contrast to previous studies, which demonstrate that rCHF can be a poor prognostic indicator in dogs with PH, although these patients were not infested with AV. Tricuspid regurgitation maximum pressure gradient was used as the sole Doppler echocardiographic parameter to quantify PH, and its lack of association with prognosis supports the consensus that assessing the wider clinical picture and response to treatment is a better assessment of the progression of the disease than a single echocardiographic parameter (Reinero et al., 2020). Age was the only other factor that had a statistically significant association with the long‐term survival of these patients, with those less than the median age of the population (43 months) having a longer survival time than those over the median age. This may be related to the chronicity of the disease in older patients, but also due to other age‐related reasons unrelated to PH and AV.

The visualisation of tricuspid regurgitation on colour Doppler can disappear in cases of AV and PH following successful treatment. However, a high‐velocity TR jet is only one marker of PH, and other markers can be considered, and the overall clinical picture should be included in the general assessment of response to treatment. There are cases where PH severity appears to be severe, based on echocardiographic variables, but the patient is coping well at home with excellent demeanour and no overt cardiorespiratory signs, supporting our previous finding that the severity of TR Max PG is not indicative of long‐term survival.

A key finding of the present study was that patients with AV‐induced PH can have a survival time of over 2 years. Indeed, while they may have prolonged hospitalisation, survival to discharge was 92.9%. Previously, only a few case reports have observed normalisation of PH after AV (Estèves et al., 2004). The time in hospital was variable, ranging from 1 to 14 days. This is likely related to oxygen dependency and severity of disease; however, the length of hospitalisation could have been affected by other factors, such as clinicians’ confidence in discharge and client‐related decisions. Owners should be advised that prolonged hospitalisation can be expected in these cases.

In terms of survival, 73.1% were known to be alive at 1 month post discharge. This is similar to a previous study where the survival for dogs with AV and bleeding diathesis 1 month post discharge was 76.9%, and for dogs with AV without bleeding diathesis was 94.8% (Thomsen et al., 2024).

Overall, it was suggested that AV and PH were the cause of death in five patients, showing AV and PH can be fatal despite hospitalisation and treatment (Koch & Willesen, 2009; Traversa et al., 2008).

### Limitations

The retrospective nature of the study means that not all information was available nor was clinical reasoning available for case management. There was no control for factors that could confound outcome. The dose and course duration of both fenbendazole and sildenafil were highly variable. Also, there was variability in the number of echocardiograms and the time between scans.

The overall population size was also small, making it difficult for further meaningful data analysis and potentially predisposing to Type II error. There was a lack of known date of deaths for more than 50% of the patients and therefore no median survival time for patients with this disease process could be extrapolated. The patients presenting to this hospital may be subject to some referral bias (Bartlett et al., 2010) and thus are not reflective of the disease process in the general canine population.

As previously mentioned, TR Max PG is only one measure of PH. The other anatomical features of PH from the ACVIM consensus statement were not used, making assessment of risk of PH and remodelling features limited to one single parameter. In addition, the Doppler assessment measure of tricuspid regurgitation velocity is user‐ and angle‐dependent; however, echocardiograms were performed by cardiology specialists or specialists‐in‐training.

This study is the first to show that dogs diagnosed with AV and PH can live for 2 years post discharge and that survival was positively associated with treatment with sildenafil. Presence of rCHF was not associated with a poorer outcome. Additionally, the study showed that dogs with PH and AV are likely to present with respiratory signs and can have prolonged hospitalisation; however, survival to discharge was favourable at 92.9%. Long‐term survival was documented, with 54.5% of patients still known to be alive at 2 years post discharge. Tricuspid regurgitation visualised by colour Doppler echocardiography resolved in nine cases. These data support conducting larger prospective studies to understand the influence of these different factors on the disease process.

## Author contributions

**R. Turner:** Investigation; writing – original draft; writing – review and editing; formal analysis. **D. Brodbelt:** Writing – review and editing; visualization; data curation; formal analysis. **D. Connolly:** Writing – review and editing; methodology; supervision. **S. Cortellini:** Writing – review and editing; supervision; formal analysis.

## Conflict of interest

The authors declare no conflicts of interest.

## Data Availability

The data that support the findings of this study are available from the corresponding author upon reasonable request.
